# 

**Published:** 1888-10-20

**Authors:** 


**To Residents, Secretaries, and Matrons.**

The Editor will be glad to have the Regulations and each Report as issued by Asylums, Hospitals, Convalescent and Nursing Institutions, as well as those of other Charities, and an early copy in duplicate of all Appeals and Festival Notices, etc., addressed to *The Editor, The Lodge, Porchester Square, London, W.* Any superintendent, secretary, matron, or other chief official who regularly sends such information may be registered, on application to the Publisher, to receive free of cost a cover for binding The Hospital in half-yearly volumes.

**Notice to Advertisers and Subscribers.**

All Advertisements and Business Communications should be addressed to The Publisher, *not to the Editor*, The Hospital, 38, Old Jewry, E.C. A Scale of Charges will be found in the Advertisement Sheets, page iii.

### Amusements and Relaxation.

### NEW PUZZLE PRIZE SCHEME.

COMMENCED OCTOBER 6th, ENDS DECEMBER 29th, 1888.

On October the 6th a New Prize Scheme was commenced in these columns.

Prizes will be awarded for the best Original Puzzles in Prose or Verse, relating to any of the Social, Philanthropic, or Political topics of the day, and taking the form of Double Acrostics, Anagrams, Square Words, Decapitations, Transpositions, or any other Puzzles which the ingenuity of the competitors may suggest. (*Answers must accompany contributions.*)

*Readers* are requested to send in weekly the Number of Marks they consider each composition to be entitled to, three being the highest score for any one contribution.

Eight Prizes of Standard Books will be given, the choice of the book to be left to the winner.

First, Second, Third, Fourth, and Fifth Prizes for best Original Puzzles, of £1 1*s.*, 15*s.*, 10*s.* 6*d.*, 7*s.* 6*d.*, and 5*s.* each.

First, Second, and Third Prize for Solutions of 15*s.*, 10*s.* 6*d.*, and 5*s.* each.

N.B.—All contributions must reach the office not later than the *First Post* on *Thursday* in each week.

### PUZZLES FOR SOLUTION.

No. 1. Double Acrostic

Dying, yet still I live,

New tints to Nature give;

Change and decay are mine—

Mine by decree divine.

Finals my primals hate,

Primals are finals fate,

Each one and all;

Whene'er my first draws near,

Flutter my last with fear—

Flutter and fall.

1. Creation of a poet's brain!
A fairy in a mortal's train,
Spellbound by witch—by mortal freed,
Supplies his master's utmost need.

2. Once was I soulless, now my love
A soul bestows; and yet I move
In fear, lest any angry mood
Should plunge me soulless in the flood.

3. Queens, Popes, and ladies all,
In pageant, state, or ball,
Their heads adorn with me,
And move complacently.

4. Two neighbour letters, once of yore
Used equally by learned lore.

5. When horror's gloom and deathly dawn

Darken the stage or tragic page;

When lightnings flash and thunders crash,

When mortals fear and ghosts appear,

Thy hand is seen, majestic queen.

6. An artist's sign of grace divine. Padre.

No. 2. Decapitation and Transposition.

Belonging to an ancient robber band,
Transposed my prey is ready found to hand.
Behead and see a witness to my deed,
Transposed it will descry my murderous greed.
Lady Clara.

No. 3. Original Anagram.

"No help in sadness, nor if unfortunate?" Tinie.

**Marks.**

| Names. | No. of Puzzles sent in Oct 11th. | No. of Marks Oct. 11th. |
|---|---|---|
| Patience | 2 | 5 |
| Lady Clara | 1 | 3 |
| Tinie | 2 | 5 |
| Bijou | 1 | 3 |
| Thanet | 3 | 7 |
| G. D. | 1 | 3 |
| Daisy | 1 | 2 |
| Salford | 3 | 6 |
| Padre | 1 | 3 |

**Notice to Correspondents.**

Doubtless owing to its being the first week of the New Competition, only a comparatively few puzzles have been received. We trust that each week will bring its full complement of puzzles, as the prizes which are offered, and the varied range of subjects suggested, will, we hope, prove attractive and easy incentives to puzzle production.

**Conditions.**

I. Every paper sent in must be clearly written, and one side only must be used. All competitors must mark at the head of their papers the number of their competition.

II. Each paper must be signed by the author with his or her real name and address. A *nom de plume* may be added if the writer does not desire to be referred to by us by his real name. In the case of all prizewinners, however, the real name and address will be published.

III. All communications relating to prize competitions should be addressed to "The Prize Editor, The Hospital, 38, Old Jewry, London, E.C." Competitors are requested not to include in letters, etc., to the Prize Editor communications on matters of other business. All letters must be fully prepaid.

IV. The Prize Editor of The Hospital is the sole judge of the competitions, and his decision is in all cases final and without appeal.

V. The Prize Editor reserves the right to publish any paper sent in, whether it receives a prize or not. He does not hold himself responsible for any MSS. sent, nor can he undertake to return rejected competitions.

